# Chemical Cross-Linking Stabilizes Native-Like HIV-1 Envelope Glycoprotein Trimer Antigens

**DOI:** 10.1128/JVI.01942-15

**Published:** 2015-12-30

**Authors:** Torben Schiffner, Natalia de Val, Rebecca A. Russell, Steven W. de Taeye, Alba Torrents de la Peña, Gabriel Ozorowski, Helen J. Kim, Travis Nieusma, Florian Brod, Albert Cupo, Rogier W. Sanders, John P. Moore, Andrew B. Ward, Quentin J. Sattentau

**Affiliations:** a The Sir William Dunn School of Pathology, The University of Oxford, Oxford, United Kingdom; b Department of Integrative Structural and Computational Biology, IAVI Neutralizing Antibody Center, Collaboration for AIDS Vaccine Discovery, and Center for HIV/AIDS Vaccine Immunology and Immunogen Discovery, The Scripps Research Institute, La Jolla, California, USA; c Department of Medical Microbiology, Academic Medical Center, University of Amsterdam, Amsterdam, The Netherlands; d Department of Microbiology and Immunology, Weill Medical College of Cornell University, New York, New York, USA

## Abstract

Major neutralizing antibody immune evasion strategies of the HIV-1 envelope glycoprotein (Env) trimer include conformational and structural instability. Stabilized soluble trimers such as BG505 SOSIP.664 mimic the structure of virion-associated Env but nevertheless sample different conformational states. Here we demonstrate that treating BG505 SOSIP.664 trimers with glutaraldehyde or a heterobifunctional cross-linker introduces additional stability with relatively modest effects on antigenicity. Thus, most broadly neutralizing antibody (bNAb) epitopes were preserved after cross-linking, whereas the binding of most weakly or nonneutralizing antibodies (non-NAb) was reduced. Cross-linking stabilized all Env conformers present within a mixed population, and individual conformers could be isolated by bNAb affinity chromatography. Both positive selection of cross-linked conformers using the quaternary epitope-specific bNAbs PGT145, PGT151, and 3BC315 and negative selection with non-NAbs against the V3 region enriched for trimer populations with improved antigenicity for bNAbs. Similar results were obtained using the clade B B41 SOSIP.664 trimer. The cross-linking method may, therefore, be useful for countering the natural conformational heterogeneity of some HIV-1 Env proteins and, by extrapolation, also vaccine immunogens from other pathogens.

**IMPORTANCE** The development of a vaccine to induce protective antibodies against HIV-1 is of primary public health importance. Recent advances in immunogen design have provided soluble recombinant envelope glycoprotein trimers with near-native morphology and antigenicity. However, these trimers are conformationally flexible, potentially reducing B-cell recognition of neutralizing antibody epitopes. Here we show that chemical cross-linking increases trimer stability, reducing binding of nonneutralizing antibodies while largely maintaining neutralizing antibody binding. Cross-linking followed by positive or negative antibody affinity selection of individual stable conformational variants further improved the antigenic and morphological characteristics of the trimers. This approach may be generally applicable to HIV-1 Env and also to other conformationally flexible pathogen antigens.

## INTRODUCTION

HIV-1, the cause of AIDS, is responsible for a pandemic of 35 million infections with more than 2 million new ones occurring each year. A prophylactic vaccine would reduce or eliminate the global spread of HIV-1, but its design has been challenging (1–4). Neutralizing antibodies (NAbs) infused into macaques or humanized mice mediate sterilizing immunity against immunodeficiency virus challenge, providing robust proof of concept for the development of a vaccine designed to elicit NAbs (1, 5, 6). Some support for antiviral, although nonneutralizing, antibody efficacy against HIV-1 comes from the phase III RV144 trial (7). The only target of antiviral antibodies is the Env complex on the virus surface, which is a noncovalently linked trimer of gp120 (surface glycoprotein) and gp41 (transmembrane glycoprotein) heterodimers. Initial attempts to engineer soluble HIV-1 Env trimers for vaccine use failed to create antigenically and morphologically correct forms (8). Tested in animal models, these nonnative proteins elicit predominantly nonneutralizing antibodies (non-NAbs) and NAbs that are active against only neutralization-sensitive (tier 1) viruses (5, 9, 10). Most clinically relevant viral isolates are relatively resistant to antibody-mediated neutralization and are classified as tier 2 or tier 3 (11). The primary goal of an Env-based vaccine is to induce antibodies that can counter such viruses.

Soluble recombinant Env trimers such as BG505 SOSIP.664, here termed BG505 trimers, have been designed that are antigenically very similar to the native, membrane-anchored Env spike; these trimers bind most broadly neutralizing antibodies (bNAbs) but few non-NAbs (12, 13). The SOSIP.664 trimers are proteolytically cleaved to produce the prefusion gp120 and gp41 forms but are engineered to contain a disulfide bond that links gp120 to gp41 covalently and a gp41-stabilizing substitution, I559P. Together, these changes prevent trimer dissociation (12). In addition, most of the gp41 membrane-proximal external region is deleted to reduce aggregation, resulting in a native-like soluble trimer that is antigenically and morphologically similar to functional membrane-anchored Env (8, 12, 13). BG505 trimers induce NAbs against the autologous tier 2 virus (14), something that has not been achieved using earlier trimer designs. Membrane-anchored native Env trimers can also induce NAbs able to neutralize autologous tier 2 viruses (15), reinforcing the importance of presenting a correctly folded form of Env to B cells. Although thermodynamically stable in solution, the BG505 trimer samples different conformations (16–18), which may reduce B-cell recognition via an immune evasion strategy termed “conformational masking” (19). For example, Env conformational flexibility can transiently expose an immunodominant structure, the gp120 V3 region (20), which elicits tier 1 NAbs and that may act as a decoy that deflects the adaptive immune response away from more immunorecessive bNAb epitopes (20, 21). Moreover, BG505 trimers engage CD4 *in vitro*, leading to the exposure of CD4-induced non-NAb epitopes (12, 22). Major goals in HIV-1 Env vaccine design are to prepare soluble trimers that expose only bNAb and not non-NAb or tier 1 V3 epitopes and that are conformationally and structurally stable.

Chemical cross-linking of HIV-1 Env was first analyzed in the context of Env proteins present on the surface of infected T cells; the outcome was that while some epitopes were destroyed, others were only modestly modified, including ones for bNAbs such as IgG1b12 (23). Glutaraldehyde (GLA) cross-linking of soluble gp120 or gp140 proteins, or membrane-anchored gp160, was found to stabilize individual conformational species (conformers) (24, 25). In an earlier study, we showed that GLA cross-linking of the nonnative uncleaved gp140_CN54_ trimer reduced its conformational flexibility, improved its antigenicity, and steered antibody responses induced in rabbits toward tier 1 NAb epitopes associated with the CD4 binding site (CD4bs) (26). A very recent study showed that when the membrane-expressed Env trimer was cross-linked *in situ*, it could be solubilized and depleted of unwanted antigenic forms by negative selection using affinity chromatography with non-NAb (27).

Here, we have cross-linked BG505 trimers with two different cross-linking agents, carried out a comprehensive antigenic and morphological analysis, and used antibody affinity columns to positively select favorable antigenic forms or negatively deplete unwanted forms. Key findings were confirmed using a second native-like SOSIP.664 trimer based on the clade B genotype B41. Overall, we provide proof of principle for the enhanced stabilization of soluble native-like trimers and the isolation of specific conformers suitable for testing as immunogens in animals.

## MATERIALS AND METHODS

### Proteins and antibodies.

Antibodies b12 (28), NIH45-46 (29), VRC01, VRC03 (30), 412D (31), A32 (32), C11 (33), CH01 (34), PGT145 (29), 2G12 (35), PGT121 (29), PGT128 (29), PGT135 (29), 14E, 19b, 39F (36), 35O22 (37), 3BC176, 3BC315 (38), PGT151 (39), CAP256-VRC26.08 (40), PDGM1400 (41), and 7B2 (42) were expressed in freestyle 293F cells under serum-free conditions and purified by protein A chromatography as previously described (43). Soluble CD4 (sCD4) (44), CD4-IgG2 (45), 15e, F105, 17b (46), PG16 (47), and b6 (28) were from the IAVI Neutralizing Antibody Consortium. Antibodies HGN194, HR10, and HJ16 (48) were a kind gift from D. Corti and A. Lanzavecchia. Antibodies were biotinylated using EZ-link NHS-LC-biotin according to the manufacturer's instructions (Fisher Scientific) and were attached to cyanogen bromide-activated agarose in accordance with the manufacturer's protocol (GE Healthcare).

BG505 SOSIP.664 (49) and B41 SOSIP.664 (50) gp140 proteins were expressed in stable CHO or 293T cells and purified as previously described, except that buffers devoid of primary amines were used. Briefly, proteins were bound to a 2G12 column, eluted with 3 M MgCl_2_, and immediately buffer-exchanged twice into 20 mM HEPES supplemented with 150 mM NaCl, followed by one buffer exchange into phosphate-buffered saline (PBS; Fisher Scientific). Alternatively, a PGT145 column was used for trimer isolation instead of 2G12, as previously described (50). The eluted trimers were concentrated and purified by size exclusion chromatography (SEC) on a Superdex 200 26/600 or 16/600 column (GE Healthcare) using PBS (Lonza) as the elution buffer. Trimer-containing fractions were pooled and concentrated and then passed down a protein A-agarose column (Pierce) to remove any potential contaminant human IgG eluted from the column, and the proteins were flash-frozen in liquid nitrogen and stored at −80°C until use.

### Cross-linking and antibody selection.

Unless otherwise specified, GLA cross-linking was performed as previously described (26). Briefly, BG505 or B41 trimers at 1 mg/ml in PBS were mixed with an equal volume of 15 mM GLA to yield a final concentration of 7.5 mM. After 5 min, 1 M Tris buffer, pH 7.4, was added, such that the final concentration was 75 mM. After 10 min, the protein was buffer-exchanged into PBS (for analysis) or Tris-buffered saline (TBS; for further purification).

EDC/NHS [1-ethyl-3-(3-dimethylaminopropyl)carbodiimide hydrochloride/*N*-hydroxysuccinimide] cross-linking was performed by mixing 2 M EDC with 20 mM NHS in MES (2-ethanesulfonic acid)-buffered saline (50 mM MES, 150 mM NaCl), pH 6.0. The cross-linker mix was then added to an equal volume of protein (in PBS) for 30 min, before the reaction was quenched with an equal volume of 1 M glycine (pH 7.4) for 10 min. The subsequent buffer exchange procedure was that used for GLA-modified proteins. The success of the cross-linking procedures was confirmed by reducing SDS-polyacrylamide gel electrophoresis (SDS-PAGE) analysis using the NuPAGE system (Life Technologies) according to the manufacturer's instructions and as previously described (26).

For immunoaffinity purification, cross-linked proteins were incubated with immobilized antibodies on columns overnight at 4°C. Unbound protein (negative selection) was washed off with TBS, and bound protein (positive selection) was eluted with 3 M MgCl_2_ and buffer-exchanged into TBS. All proteins were sterile-filtered with Costar Spin-X 0.22-μm filters and either stored at 4°C or flash-frozen in liquid nitrogen and stored at −80°C. Proteins were analyzed by reducing SDS-PAGE as previously described (26).

### ELISA. (i) Standard capture ELISA.

For standard capture ELISA, ELISA plates (Greiner Bio-One) were coated with 4 μg/ml of capture MAb 2G12 at 4°C overnight in PBS. After blocking with 2% bovine serum albumin (BSA) in PBS–0.05% Tween, BG505 trimers (0.2 μg/ml) were captured and then labeled with a range of concentrations of biotinylated antibodies, followed by peroxidase-conjugated streptavidin. The colorimetric endpoint was obtained using the 1-Step ultra tetramethylbenzidine (TMB) substrate (Thermo Scientific) until a signal of approximately 1.0 unit of optical density at 450 nm (OD_450_) was generated for each antibody. Color development was stopped with sulfuric acid (0.5 M), and the OD_450_ was measured. In general, substrate development times were substantially longer for non-NAbs than for bNAbs, reflecting the more limited exposure of the former class of epitopes. For this reason, OD_450_ values for the binding of non-NAbs and bNAbs cannot be directly compared.

### (ii) Inverted capture ELISA.

We observed a high background signal when biotinylated V3 MAbs were used for detection. We therefore established an inverted ELISA format in which a dilution series of BG505 trimers was captured by plate-immobilized V3 antibody 19b and detected using biotinylated 2G12 and peroxidase-conjugated streptavidin as described above. Both conventional 2G12 capture and inverted ELISAs were used to confirm that trimer forms with exposed V3 regions had been depleted by the negative-selection affinity columns.

### (iii) Direct antigen coating ELISA.

For direct antigen coating ELISA, trimers were coated at 2 μg/ml in PBS directly onto the plate overnight at 4°C without antibody capture. Subsequent steps for blocking, primary and secondary antibody binding, and substrate development were performed as described above and previously (26).

All ELISA signals were corrected by subtracting the background signal obtained in the absence of both antigen and primary antibody, and the resulting data were plotted using GraphPad Prism V6.0. To generate binding indices from ELISA titration curves, an area under the curve (AUC) analysis of ligand-trimer binding was performed; the binding index represents the ratio of cross-linked trimer value to the value of the matched unmodified trimer that was used for cross-linking. Binding indices were calculated as [AUC(mod)−AUC(blank)]/[AUC(unmod)−AUC(blank)], where “mod” represents modified (cross-linked and positively selected as stated), “unmod” represents unmodified, and “blank” represents the negative-control curve of the respective MAb without antigen. Indices of <1 indicate reduced binding to the cross-linked trimer compared to that of its unmodified counterpart, and the converse for values of >1. Statistical analysis was performed in Prism using the tests indicated in the various figure legends.

### SPR.

Surface plasmon resonance (SPR) experiments were performed using a Biacore 3000 instrument (GE Healthcare), essentially as previously described (26). Briefly, a sufficient amount of each test antibody to generate a signal of ∼700 response units (RU) was captured onto the flow cell of a CM5 chip via immobilized anti-human IgG antibodies at 37°C. BG505 trimers (5 μg/ml) were then passed over immobilized antibodies at 30 μl/min for 5 min, followed by a 5-min dissociation phase. Data were normalized by subtracting signal from an irrelevant control antibody followed by subtraction of an equally processed buffer injection (double referencing). Subsequently, data were adjusted for minor fluctuations in antibody capture levels by dividing by the actual signal (of response before trimer injection minus baseline response before capture) and multiplying it by the target immobilization level (700 RU). Data were plotted using Prism.

### DSC.

Thermal denaturation of native and cross-linked BG505 was studied using a nano-differential scanning calorimetry (nano-DSC) calorimeter (TA Instruments). All Env protein samples were first extensively dialyzed against PBS, and the protein concentration was then adjusted to 0.1 to 0.3 mg/ml. After the sample was loaded into the cell, thermal denaturation was probed at a scan rate of 60°C/h to a maximum temperature of 80°C for non-cross-linked and 100°C for cross-linked BG505. Buffer correction, normalization, and baseline subtraction procedures were applied before the data were analyzed using NanoAnalyze software v.3.3.0 (TA Instruments). The data were fitted using a non-two-state model, as the asymmetry of some of the peaks suggested that unfolding intermediates were present. We report the main thermal denaturation (*T_m_*) values in the text of this article, while the multiple *T_m_* values are in the supplemental material.

### Negative-stain EM. (i) Sample preparation.

Samples were analyzed by negative-stain electron microscopy (EM). A 3-μl aliquot containing ∼0.05 mg/ml of the trimer was applied for 25 s onto a carbon-coated 400-mesh Cu grid that had been glow discharged at 20 mA for 30 s. The grid was then negatively stained with 2% uranyl formate for 30 s. Data were collected using a FEI Tecnai Spirit EM operating at 120 kV, with an electron dose of ∼29 e^−^/Å^2^ and a magnification of ×52,000 that resulted in a pixel size of 2.05 Å at the specimen plane. Images were acquired with a Tietz 4k × 4k TemCam-F416 CMOS camera using a nominal defocus of 1,000 nm and the Leginon package (51).

### (ii) Data processing.

Particles were picked automatically using DoG Picker and put into a particle stack using the Appion software package (52, 53). Initial, reference-free, two-dimensional (2D) class averages were calculated using particles binned by two via the iterative MSA/MRA clustering 2D alignment (54) and IMAGIC softwares (55). To analyze the quality of the trimers (closed native trimers, open native trimers, or nonnative trimers), the reference-free 2D class averages were examined by eye using the metrics described previously (50).

### Computational modeling of modification sites.

The crystal structure of the BG505 trimer (Protein Data Bank [PBD] accession no. 4TVP) was fitted into the electron density of membrane-bound Env (Electron Microscopy Data Bank [EMDB] maps 5019 and 5022) using UCSF Chimera 1.10.1 (56). Crystal structures of the indicated antibody-epitope complexes were added by superimposing the epitope onto the BG505 trimer. Trimer apex antibodies for which no structural analysis has been reported to date were aligned using published electron density for PG9 in complex with BG505 gp140 (EMDB map 2241). BG505 trimer lysine residues within 8 Å of an antibody epitope were defined as being in close proximity with potential for epitope modification by GLA. The electron density of the membrane-bound trimer was colored according to the extent of overlap with the indicated aligned antibody crystal structures and rendered using the Chimera program.

## RESULTS

### Optimizing GLA cross-linking conditions for BG505 SOSIP.664 trimers.

**FIG 1:**
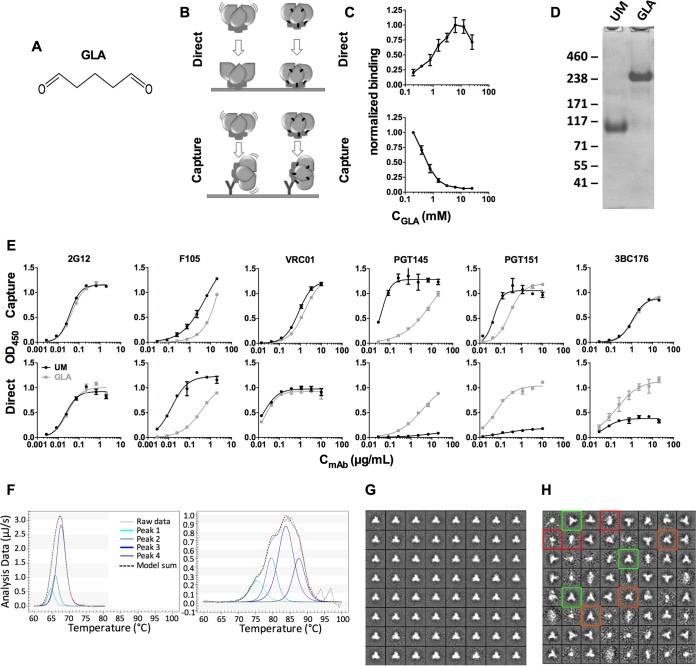
Direct coating ELISA and establishment of optimal cross-linking conditions. (A) Structure of the GLA cross-linker. (B) Depiction of the ELISA formats: 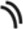 indicates conformational instability, and black dashes indicate GLA cross-links within the trimer. (C) ELISA data corresponding to panel B; PGT145 binding to BG505 trimers cross-linked by different GLA concentrations and assayed by direct binding or capture ELISA. (D) Reducing SDS-PAGE analysis of BG505 trimers, either unmodified (UM) or cross-linked with 7.5 mM GLA for 5 min. (E) Unmodified and GLA cross-linked trimers were analyzed for antibody binding by direct coating and capture ELISA. Color development times differ for each antibody and between the ELISA formats to yield comparable endpoint signals, and hence the plots are not representations of the relative exposure of different epitopes on the trimers. Each datum point represents the mean of triplicates ± standard error of the mean (SEM). Data are representative of at least two independent experiments. (F) Unmodified and GLA cross-linked material was analyzed by DSC. Modeled peaks from a non-two-state fit as well as their sum are indicated. Data from the DSC analysis are listed, including individual peaks of a non-two-state model. (G and H) Negative-stain EM analysis of unmodified and GLA-treated BG505 trimers. 2D class averages are shown for unmodified (G) and GLA cross-linked (H) trimers. Exemplary closed native-like trimers (green squares), open native-like trimers (orange squares), and nonnative-like trimers (red squares) are marked in panel H. In total, >95% of trimers in panel G were correctly folded, whereas 2,466 of 4,561 (54%) of the GLA cross-linked trimers in panel H were correctly folded.

We have reported that cross-linking soluble, uncleaved, nonnative gp140 proteins with glutaraldehyde (GLA), a bifunctional aldehyde that cross-links amine groups primarily associated with lysine side chains (57), prevented dissociation of gp140 subunits under the harsh denaturing conditions that apply during reducing SDS-PAGE but preserved the epitopes of many NAbs (26) (Fig. 1A). However, reducing SDS-PAGE does not reveal the stability of individual bNAb epitopes under milder and more physiologically relevant denaturing conditions. To explore the effects of cross-linking on native-like BG505 SOSIP.664 trimers, we first established an ELISA based upon direct binding of the trimer to the solid phase, which exposes the glycoprotein to hydrophobic and electrostatic interactions that may perturb its conformation, and compared the results to a 2G12 antibody capture ELISA format in which the trimer conformation is preserved (12) (Fig. 1B). Since the D7324 epitope tag is not compatible with some cross-linking chemistries such as EDC (see below), we captured the trimers with the bNAb 2G12 that binds to gp120 glycans in a manner that is not influenced by any of the cross-linking conditions used (data not shown). Steric clashes between 2G12 and the various detection antibodies are at most minimal in the trimer context (13). The results obtained when D7324 or 2G12 was used to capture D7324-tagged, GLA-cross-linked BG505 trimers were highly concordant (Pearson *r* = 0.97, *P* < 0.0001; data not shown).

After completing the above exploratory studies, we used the direct binding and indirect capture (2G12) ELISA formats and reducing SDS-PAGE analysis to optimize various cross-linking parameters. We used the quaternary epitope-specific bNAb PGT145 to assess trimer folding, as it is a highly stringent probe for native conformation. Accordingly, we identified the optimal GLA concentration (7.5 mM), PBS buffer pH (7.4), and quenching conditions (75 mM Tris) that best preserved the PGT145 epitope on the 2G12-captured (Fig. 1C and data not shown) and directly coated (Fig. 1C and E) trimers, while also stabilizing the trimer by minimizing its dissociation into dimers and monomers after reducing SDS-PAGE (Fig. 1D). The optimized cross-linking conditions nevertheless did reduce PGT145 binding to cross-linked trimers compared to unmodified BG505 trimer by 3.7-fold. Antigenicity analysis of unmodified trimers compared to those cross-linked with GLA under the optimal conditions defined above revealed that direct coating of trimers onto ELISA wells perturbed several quaternary bNAb epitopes (PGT145, PGT151, 3BC176) but that cross-linking reduced the extent to which this occurred (Fig. 1E). Comparing the results from the two ELISA formats showed that the conformationally insensitive, mannose-specific gp120 epitope for the 2G12 bNAb and the CD4bs epitope for the VRC01 bNAb were essentially unaffected by direct coating of the trimers. However, the F105 non-NAb bound more strongly to its nonneutralizing CD4bs epitope when the untreated trimers were directly coated than when captured indirectly, an outcome consistent with a partial unfolding of the native trimers when they associate directly with the solid phase (Fig. 1E). GLA cross-linking reduced F105 binding in both assay formats.

As measured by differential scanning calorimetry (DSC), cross-linking increased the melting temperature of the trimer by ∼16°C from 67.4°C to 83.8°C for the main transitions (Fig. 1F and Table 1). In addition, the partial unfolding that occurred over an extended temperature range suggests there is heterogeneity within the population of cross-linked trimers. Such heterogeneity is expected, as cross-linking will trap a variety of transient BG505 conformational forms, as previously shown for other Env antigens (24, 25). To assess whether there is visible morphological heterogeneity within the population of cross-linked trimers, we performed a negative-stain EM analysis. As observed previously (8, 12), unmodified BG505 trimers purified by 2G12 affinity chromatography and SEC were predominantly (>95%) in a correctly folded form (Fig. 1G). In contrast, only ∼50% of the GLA-treated trimers adopted a native-like “closed” conformation, the rest having a visibly modified morphology (Fig. 1H) that is probably related to the heterogeneity observed by DSC. Nevertheless, since the quaternary epitope-specific bNAbs (with the exception of PGT145) bound similarly to the unmodified and GLA cross-linked trimer populations, we can infer that any structural variation imposed by cross-linking must be limited in its extent. Taken together, the EM analysis shows that cross-linking affects the conformational integrity of about half of the trimers, which may account for the partial reduction in the binding of some of the antibodies described above.

**TABLE 1:** DSC analysis of cross-linked BG505 trimers

Sample ID^*a*^	*T_m_* of individual peak(s) (°C)	Onset temp (°C)	Width of peak (°C)
Unmodified	64.4, 65.7, 67.4	∼62	∼10
GLA	75.4, 79.5, 83.8, 87.6	∼70	∼22
GLA + PGT151	85.0	∼72	∼22
EDC/NHS	77.2, 83.0, 86.1, 90.3	∼72	∼22

a ID, identifier.

In summary, the optimal GLA cross-linking procedure partially preserves the appropriate morphology of the BG505 trimers, including the binding of PGT145 to its quaternary epitope. The procedure also maintains bNAb binding to other quaternary and nonquaternary epitopes under the mildly denaturing conditions that arise when trimers are coated directly onto ELISA wells, conditions that otherwise adversely influence the conformation of the trimers.

### Antigenic profile of GLA cross-linked BG505 trimers.

**FIG 2:**
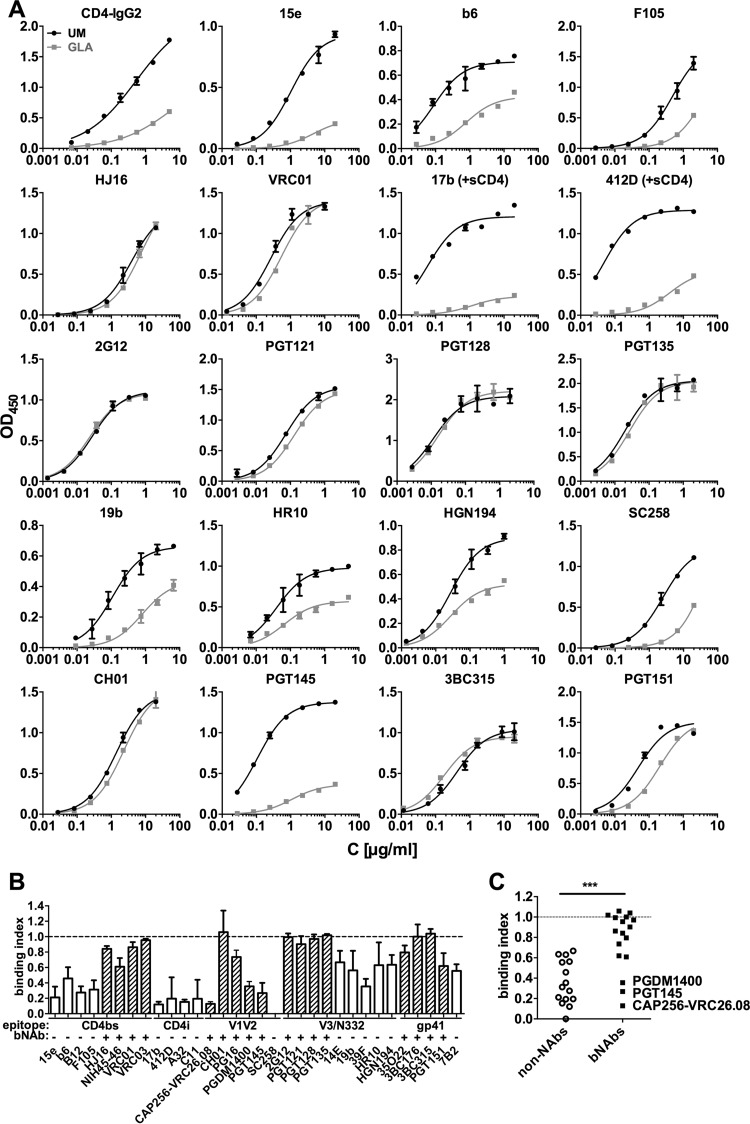
GLA cross-linking of BG505 trimers. The trimers were cross-linked with 7.5 mM GLA, and their antigenicity was tested by capture ELISA. (A) Representative titration curves from a capture ELISA are shown for selected antibodies. Error bars indicate the SD of results from replicate wells. Data are representative of two to six independent experiments. (B and C) Binding indices were calculated as AUC (cross-linked)/AUC (unmodified). Values of <1 indicate that there was a reduction in antibody binding; values of 1 (dotted line) represent no change in binding. (B) Mean binding indices from at least two independent experiments are shown for all antibodies tested. (C) The antibodies are grouped into neutralizing and nonneutralizing categories. ***, *P* < 0.001 (Mann-Whitney U test).

Based on the above-described observations, we used a larger panel of antibodies and related probes of trimer conformation to more thoroughly explore the effect of GLA cross-linking. We first used the 2G12 capture ELISA, in which the trimer conformation remains native-like, to generate binding curves (Fig. 2A) or binding indices (Fig. 2B and C; see also Table S1 in the supplemental material). The latter are based on an area under the curve (AUC) analysis of ligand-trimer binding; values of <1 indicate reduced binding to the cross-linked trimer compared to its unmodified counterpart, and the converse for values of >1. Consistent with our previous results using uncleaved, nonnative gp140 proteins (26), cross-linking had variable effects on the binding of ligands to the CD4bs on the BG505 trimers. Ligands that require conformational flexibility for optimal binding to gp120, such as CD4 (CD4-IgG2) and the CD4bs non-NAbs 15e, b6, and F105, (17, 19, 58–61), and that bind only weakly to the native BG505 trimer (58) bound even less well (by 2- to 5-fold) to the cross-linked trimers (Fig. 2A and B). Note that ELISA color development times were extended to compensate for the weak binding of non-NAbs, meaning that non-NAb and bNAb OD values are not directly comparable. In contrast, CD4bs bNAbs that do not require extensive conformational flexibility and that recognize the ground state of the trimer (VRC01, VRC03, HJ16, and NIH45-46) (16, 17) were minimally affected by cross-linking (1.1- to 1.6-fold reduction in binding) (Fig. 2A and B). The recognition of CD4-induced (CD4i) epitopes for non-NAbs 17b, 412D, A32, and C11 in the presence of sCD4 was almost completely eliminated on the cross-linked trimers, providing further evidence that these trimers were now largely conformationally static. Cross-linking did not affect binding of the glycan-dependent bNAbs to the mannose patch (2G12) or N332-glycan supersite (PGT121, PGT128, PGT135) epitopes, which is again consistent with these bNAbs not requiring conformational changes to recognize the trimer (62). Non-NAbs (for BG505) 19b, HR10, 39F, and HGN194 against the V3 region bound the unmodified trimers efficiently in capture ELISA, as previously reported (12, 13), but less so (1.5- to 3-fold reduction) to their cross-linked counterparts (Fig. 2A to C). In addition, the binding of the V2-specific non-NAb SC258 was almost completely eliminated after cross-linking. The quaternary epitopes at the apex were variably affected by trimer cross-linking: binding of PGT145, PGDM1400, and CAP256-VRC26.08 was substantially (3.7-, 2.8- and 7.8-fold, respectively) reduced, consistent with the data shown in Fig. 1, whereas CH01 and PG16 binding levels were essentially unaffected. The bNAbs 35022, 3BC176, 3BC315 (63), and PGT151 bound similarly to their quaternary epitopes at the gp120-gp41 interface on both the unmodified and cross-linked trimers.

When the binding indices for non-NAbs and bNAbs were analyzed by group (Fig. 2C), the reduction in non-NAb binding to the cross-linked trimers was highly significant compared to the bNAb group (*p* = 0.0002, Mann-Whitney U test). Among the bNAbs, only PGT145, PDGM1400, and CAP256-VRC26.08 to V1V2 quaternary epitopes bound >2-fold less well to the cross-linked trimers than the unmodified ones. Possible explanations are that the conformation of the trimer apex may be adversely affected by aldehyde cross-linking of amine groups (see below), that cross-linking preserves the correct folding of trimers but traps conformational variants that display the apex epitope inefficiently or not at all, and that one or more of the lysine residues 168, 169, and 171 that are potentially contact sites for PGT145 are directly affected by GLA adduction. However, as the recognition of closely related quaternary epitopes at the trimer apex by the CH01 and PG16 bNAbs was essentially unaffected by cross-linking, either any structural impact of cross-linking on this region of the trimer may be highly localized or these two bNAbs are less reliant on lysines for binding.

As noted above, antibody binding may in principle be modified by any direct effects of GLA on lysines within, or proximal to, antibody epitopes. To evaluate this scenario, we interrogated the PDB for antibody-antigen structures that revealed lysines inside or within 8 Å of the epitope, corresponding to the approximate maximum distance between lysine side chains that could accommodate cross-linking with monomeric GLA (64). Similar to our previous results using uncleaved gp140 or membrane-expressed Env (26), there was no correlation between the position of lysine residues and the antibody binding index, even though all the antibodies except a minority of N332-requiring bNAbs bound to epitopes that included, or were in close proximity to, at least one lysine residue (Table 2). We conclude that GLA modification of lysines is not a major factor influencing epitope recognition for the analyzed antibodies, although we cannot exclude some effect on binding of antibodies for which lysines make critical contacts.

**TABLE 2:** Lysines on BG505 in proximity (<8.0 Å) of MAb epitopes

MAb	PDB accession no.	Lysine residue(s)^*a*^	Reference
F105	3HI1	121, 421	59
b12	2NY7	155, 421	79
HJ16	4YE4	282	
NIH45-46	3U7Y	97, 282	80
VRC01	3NGB	97, 282	81
17b	4JM2	117, 121	62
A32	4R4F	59, 117, 207	82
PG9	3U4E	168, 169, 171	83
PGT122	4TVP		61
PGT128	3TYG		84
PGT135	4JM2	335	62
35O22	4TVP	46	61
3BC315		655	63

a All residues are numbered according to the HxBc2 numbering scheme.

### EDC/NHS cross-linking.

**FIG 3:**
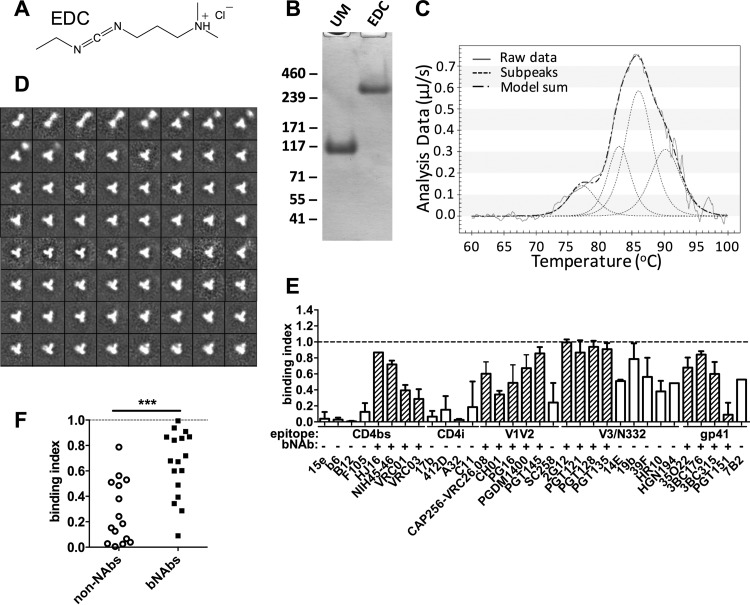
EDC/NHS cross-linking of BG505 trimers. (A) Chemical structure of EDC cross-linker. (B) Reducing SDS-PAGE gel of unmodified (UM) and EDC/NHS cross-linked (EDC) BG505. (C) EDC/NHS cross-linked material was analyzed by DSC. Modeled peaks from a non-two-state fit and their sum are indicated. (D) Negative-stain EM analysis of EDC/NHS cross-linked BG505: 2D class averages are shown. (E) EDC/NHS cross-linked BG505 was analyzed by capture ELISA. Binding indices were calculated as AUC (cross-linked)/AUC (unmodified). Values of <1 indicate there was a reduction in antibody binding; values of 1 (dotted line) represent no change in binding. Mean binding indices from at least two independent experiments are shown for all MAbs tested. (F) The MAbs are grouped into neutralizing (bNAbs) and nonneutralizing (non-NAbs) categories. ***, *P* < 0.001 (Mann-Whitney U test).

The chemical structure of GLA in aqueous solution is sufficiently heterogeneous that GLA can react with several different amino acids, among which lysine residues are the most commonly affected (reviewed in reference 57). The modification reaction attaches a carbon chain of at least 5 carbon atoms to the modified residue (Fig. 1A). As noted above, one possible consequence is that bNAb epitopes are directly affected, while another is that potentially immunogenic neo-epitopes may be created (26). An alternative approach is to use synthetic cross-linkers that have a better-defined specificity and do not leave linker atoms after cross-linking, therefore potentially better maintaining the original antigenicity of the modified protein. EDC (Fig. 3A) is a heterobifunctional reagent that cross-links amine to carboxyl groups and leaves no residual atoms (i.e., it is a “zero-length” cross-linker) (65), while NHS stabilizes the reaction. Using our direct-coating ELISA, we optimized cross-linking of BG505 trimers with EDC/NHS and established conditions that retained the PGT145 epitope substantially better than the GLA cross-linking approach (see Materials and Methods; data not shown).

Biochemically, EDC/NHS and GLA cross-linking yielded comparable results, with the resulting trimer remaining fully intact when analyzed by reducing SDS-PAGE (Fig. 3B). A DSC analysis also showed that the EDC/NHS-modified trimers were substantially more stable than unmodified trimers (∼19°C increase for the main transitions) and slightly more stable than GLA cross-linked trimers (∼3°C increase) (Fig. 3C and Table 1). Negative-stain EM imaging showed that ∼90% of the EDC/NHS-treated trimers retained a closed native-like conformation, which is consistent with the minimal reduction in PGT145 binding, with the remaining ∼10% being either partially open native-like trimers or nonnative forms (Fig. 3D). Overall, we conclude that EDC/NHS cross-linking robustly stabilizes BG505 trimers, with only ∼5% of the trimer population adopting nonnative conformations, compared to ∼50% for GLA cross-linking.

Antigenicity analysis revealed that, with some exceptions, the epitopes for most bNAbs were well conserved after EDC/NHS cross-linking. The reductions in binding ranged from none for 2G12 to 1.7-fold for 3BC315 (Fig. 3E and see Table S1 in the supplemental material). However, binding of the CD4bs bNAbs VRC01 and VRC03 was reduced by 2.5- and 5-fold, respectively. This moderate level of reduction may reflect condensation of the trimer by EDC/NHS cross-linking that further reduces an already highly restricted angle-of-approach to the CD4bs and/or magnifies steric clashes with the opposing gp120 protomer (17, 60, 66). Particularly striking were the differences in quaternary bNAb reactivity with the trimers cross-linked with GLA compared to those cross-linked with EDC/NHS. Thus, binding of the gp120-gp41 interface bNAb PGT151 was reduced by 11-fold by EDC/NHS (Fig. 3C and see Table S1 in the supplemental material) compared to only 1.5-fold by GLA (Fig. 2A and B). Moreover, whereas binding of the trimer apex bNAb PGT145 to the GLA cross-linked trimers was reduced by 3.7-fold (Fig. 2B and C), the corresponding reduction after EDC/NHS cross-linking trimers was only 1.2-fold (Fig. 3E). Other quaternary V1V2-specific bNAbs, PG16, PGDM1400, and CAP256-VRC26.08, were affected to an intermediate extent (2.0-, 1.5- and 1.7-fold reductions, respectively). In contrast, binding of CH01 to the trimer apex was reduced by 3.2-fold after EDC/NHS cross-linking, whereas GLA had no detectable effect on this epitope. Taken together, these results emphasize how localized and selective any impact of the cross-linker can be, although again it is worth emphasizing that any reductions in bNAb binding were generally <5-fold and that no epitope was completely destroyed. As with GLA, EDC/NHS cross-linking substantially reduced the binding of most non-NAbs, in particular those to CD4bs and CD4i epitopes, although again, V3 loop non-NAb reactivity was reduced to different extents (1.3- to 2.6-fold). Overall, the reduction in non-NAb binding compared to bNAb binding was highly significant after EDC/NHS cross-linking (*P* = 0.0006, Mann-Whitney U test), similar to that seen using GLA (Fig. 3F, compare with Fig. 2C).

### Positive and negative affinity selection of cross-linked BG505 trimers.

Cross-linking introduced heterogeneity into the normally homogeneous population of BG505 trimers (Fig. 1 and 3). To prepare more homogeneous populations of cross-linked trimers with native-like appearance and antigenicity, we employed positive selection using quaternary epitope-specific bNAbs as affinity chromatography reagents. Thus, GLA cross-linked trimers were passed through medium-scale immunoaffinity columns bearing PGT145 or PGT151, while their EDC/NHS cross-linked counterparts were selected using PGT145 or 3BC315 columns (PGT151 was not used in the latter case since this epitope was adversely affected by EDC/NHS cross-linking). Unbound proteins from the affinity columns were discarded, while the bNAb-bound trimers were eluted and analyzed. With the PGT151 column, 60 to 75% of the input GLA-cross-linked trimers were recovered after positive selection (*n* = 3 independent experiments), whereas with PGT145, the recovery was ∼30% (*n* = 2). The different yields are consistent with the greater loss of PGT145 binding to GLA cross-linked trimers compared to the binding of PGT151 (3.7-fold versus 1.6-fold, respectively). The percentage recovery of GLA-treated trimers from the PGT145 column is also broadly compatible with the EM analysis that showed that only ∼50% of these trimers retained their native-like structure. The yields of positively selected EDC cross-linked material were ∼60% to 70% and ∼50% to 60% for PGT145 and 3BC315 selection, respectively (*n* = 2 independent experiments).

**FIG 4:**
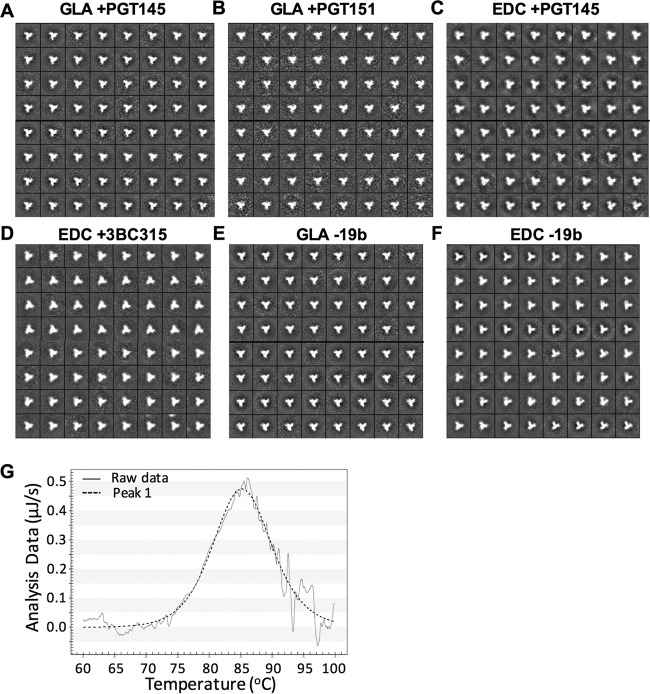
Negative-stain EM and DSC analysis of unmodified and cross-linked, antibody-selected BG505 trimers. 2D class averages are shown for GLA cross-linked and PGT145 positively selected (GLA +PGT145) (A), GLA cross-linked and PGT151 positively selected (GLA +PGT151) (B), EDC cross-linked and PGT145 positively selected (EDC +PGT145) (C), EDC cross-linked and 3VC315 positively selected (D), GLA cross-linked and 19b negatively selected (E), and EDC cross-linked and 19b negatively selected (F) trimers. (G) DSC analysis of GLA cross-linked BG505 trimers positively selected on PGT151.

We used negative-stain EM analysis to visualize the various positively selected cross-linked BG505 trimer subpopulations. In all cases, the trimers were >90% native-like in the closed conformation (Fig. 4A to D), confirming that each of the quaternary bNAbs enriched trimer populations that were properly folded. Other experiments have shown that PGT145 purification of unmodified BG505 trimers selects for or drives the formation of a “partially open” native-like trimer subpopulation (S. W. de Taeye, G. Ozorowski, A. T. de la Peña, M. Guttman, J.-P. Julien, T. L. G. M. van den Kerkhof, J. A. Burger, L. K. Pritchard, P. Pugach, A. Yasmeen, J. Crampton, J. Hu, I. Bontjer, J. L. Torres, H. Arendt, J. DeStefano, W. C. Koff, H. Schuitemaker, D. Eggink, B. Berkhout, H. Dean, C. LaBranche, S. Crotty, M. Crispin, D. C. Montefiori, P. J. Klasse, K. K. Lee, J. P. Moore, I. A. Wilson, A. B. Ward, and R. W. Sanders, submitted for publication). This effect of PGT145 affinity purification appears to be prevented by cross-linking. DSC analysis of GLA cross-linked trimers with and without PGT151 positive selection showed no substantial thermal stability differences between the populations (Fig. 4G and Table 1). Thus, the observed heterogeneity in the nonselected trimer population may result primarily from stochastic cross-linking events rather than the presence of a mixture of different conformational states that are all captured by cross-linking.

**FIG 5:**
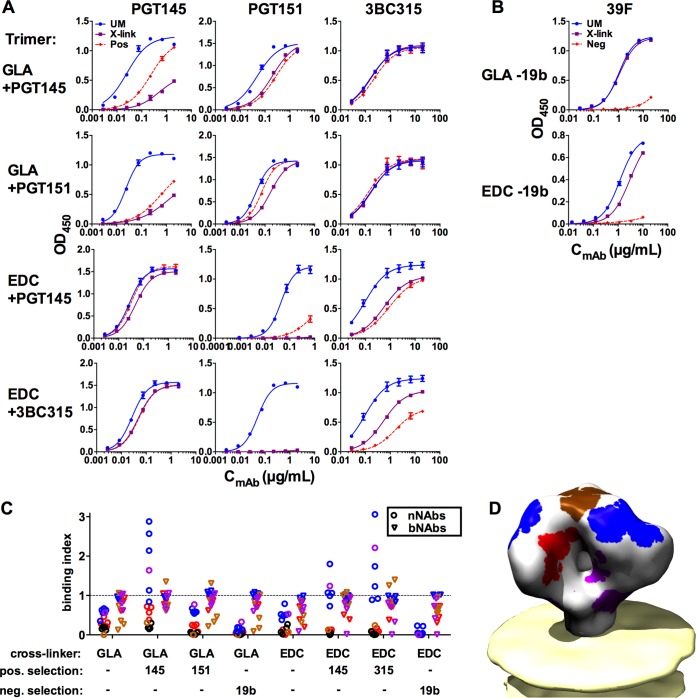
Positive and negative selection of GLA or EDC/NHS cross-linked BG505 trimers. (A and B) bNAb and non-NAb binding to positively (A, Pos) and negatively (B, Neg) selected cross-linked trimers was compared to that of unmodified (UM) and cross-linked unselected (X-link) trimers by capture ELISA. (C) Summary of changes in bNAb and non-NAb binding. Data for antibodies against the CD4bs (red), V2 loop (brown), V3 (blue), and gp41 (purple) are represented in color as indicated. Black symbols indicate other non-NAb epitopes. Values of 1 (dotted line) represent no change in binding, and values of <1 indicate reduced binding and values of >1 indicate increased binding compared to unmodified protein. (D) Model of a membrane-bound trimer (EMD-5019 and EMD-5022) with the epitopes indicated in color as described for panel C.

Antigenicity studies showed that positive selection on bNAb columns increased the binding of the enriched, cross-linked trimers to the corresponding bNAb, compared to the input (i.e., unselected) cross-linked material (Fig. 5A). For GLA cross-linked trimers, PGT145 positive selection allowed a 2.8-fold increase in PGT145 binding, compared to unselected material, although even after positive selection, PGT145 binding was modestly (1.3-fold) reduced compared to unmodified protein. In contrast, PGT151 selection resulted in trimers that bound the PGT151 similarly to unmodified trimers (Fig. 5A). Selection of EDC/NHS cross-linked trimers on a PGT145 column yielded trimers that bound to PGT145 indistinguishably from unmodified trimers that were also purified on the same column. In contrast, 3BC315 selection failed to enrich 3BC315-reactive trimers in two independent experiments, although the column flowthrough (i.e., 3BC315 nonreactive Env) did react relatively poorly with 3BC315 (Fig. 5A and data not shown). This may be the result of selected trimers binding to the column too tightly to be eluted under standard conditions.

Most other bNAbs bound comparably to the GLA or EDC/NHS cross-linked trimer populations that were enriched via the different columns (see Fig. S1A to D and Table S1 in the supplemental material), but some striking differences were observed for non-NAb binding. Thus, trimers cross-linked with either GLA or EDC/NHS and then PGT145 selected or EDC/NHS cross-linked and then 3BC315 selected had increased reactivity of 1.2-fold to >3-fold with V3 non-NAbs (see Fig. S1A to D and Table S1). Similar increases were observed for the gp41 non-NAb 7B2, implying that the columns had selected trimers with global conformational differences affecting both the gp120 and gp41 subunits. However, for PGT151-selected, GLA cross-linked trimers, V3 non-NAb binding was reduced compared to that of unmodified trimers selected on the same column. Thus, whether or not there can be an increase in V3 exposure is influenced by the selecting bNAb (see Fig. S1A to D and Table S1).

Because the V3 region is a highly variable immunodominant structure that induces limited or nonneutralizing activity against viruses relevant to transmission, we considered it important to reduce V3 exposure on cross-linked trimers. When assessed by capture ELISA, V3-specific non-NAbs bind BG505 trimers despite failing to neutralize the corresponding Env-pseudotyped virus (12). However, EM analysis shows that only a small proportion of trimers bind V3 non-NAbs, and these antibodies are only minimally trimer reactive in SPR assays (12). One explanation of the assay-dependent exposure of the V3 region is that under equilibrium conditions (as in ELISA), V3 antibodies may “trap” trimer conformers that transiently expose their V3 regions. In contrast, under more dynamic binding conditions (as in EM or SPR), such trapping is less pronounced. Since cross-linking will irreversibly “lock” all conformers within a trimer population, we hypothesized that we could use V3 non-NAb negative-selection columns to deplete V3-reactive trimers without reestablishing an equilibrium in which V3 again becomes exposed. We tested this hypothesis using both GLA and EDC/NHS cross-linked BG505 trimers. After incubation of the GLA or EDC cross-linked trimers with column-immobilized 19b, ∼55% to 75% of the protein was recovered. Similar to the outcome with positive selection, V3 non-NAb depletion yielded trimers that were >90% in the closed conformation, as judged by negative-stain EM analysis (Fig. 4E and F). ELISA reactivity of the unbound (i.e., V3-depleted) fraction with 19b and all other V3 non-NAbs tested was almost completely eliminated. For example, the maximum ELISA binding signal for the V3 non-NAbs HGN194 and HR10 was reduced by >10-fold compared to the unselected, unmodified trimer (48) (see Fig. S1E and F and Table S1 in the supplemental material). For comparison, we also studied the GLA cross-linked 19b-bound trimer subpopulation after eluting it with 3 M MgCL_2_. As expected, these trimers reacted strongly with V3 non-NAbs but poorly with quaternary epitope-specific bNAbs. Hence, these V3-exposed trimers are not in a native-like conformation (data not shown).

Thus, in summary, cross-linking globally reduces non-NAb binding to trimers while broadly preserving bNAb epitopes, and positive and negative affinity selection can further improve both the antigenic (Fig. 5, see also Fig. S1 and Table S1 in the supplemental material) and morphological (Fig. 4) properties of the resulting trimers in an antibody- and epitope-specific manner.

**FIG 6:**
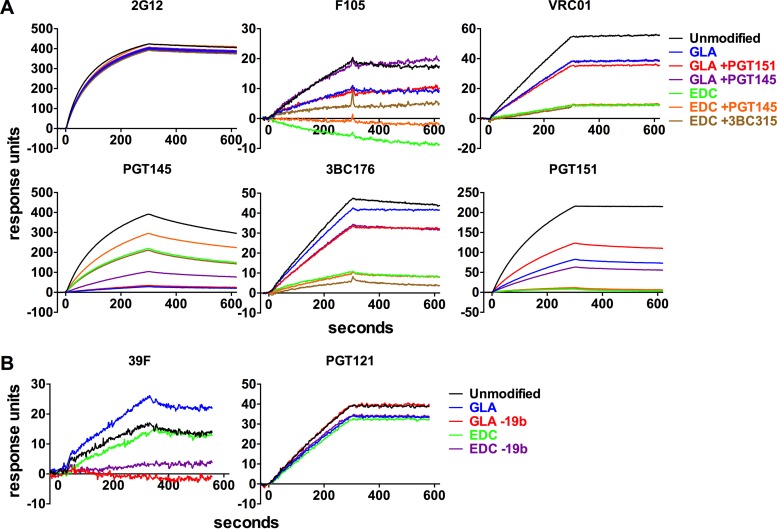
SPR analysis of positively and negatively selected trimers. (A and B) The indicated bNAb and non-Nab were captured onto Biacore sensorchip surfaces via immobilized anti-human IgG antibody (not shown). The indicated positively (A) and negatively (B) selected cross-linked trimers were passed over the surface for 5 min (starting at 0 s), followed by a 5-min dissociation period.

To further investigate the antigenicity of MAb-selected trimer subpopulations, we used SPR (Fig. 6). Unlike ELISA, SPR measures binding kinetics in real time and allows a more precise evaluation of trimer antigenicity (67). In agreement with the ELISA data, 2G12 binding levels in SPR were comparable for all the trimer subpopulations, irrespective of whether they were cross-linked or positively selected with bNAbs (Fig. 6A). The CD4bs non-NAb F105 bound only weakly to the unmodified BG505 trimer, but its reactivity was further reduced (GLA) or completely eliminated (EDC/NHS) by cross-linking. Positive selection with quaternary epitope-specific bNAbs also changed F105 binding, albeit subtly. The CD4bs bNAb VRC01 bound unmodified BG505 trimers fairly weakly and less well to trimers cross-linked with GLA or EDC/NHS. More specifically, the VRC01 binding kinetics were similar between conditions, but the maximum binding was reduced after cross-linking. Thus, there may be a subpopulation of trimers or gp120 protomers for which VRC01 binding is compromised by cross-linking. Consistent with the ELISA data, binding of the quaternary epitope bNAb PGT145 was substantially reduced by GLA cross-linking but less so by EDC/NHS, and it was increased after PGT145-positive selection of EDC/NHS cross-linked trimers. The inverse was true for 3BC176 and PGT151, for both of which binding was modestly reduced by GLA cross-linking but substantially reduced by EDC/NHS.

The effect of V3 non-NAb negative selection was analyzed using the 39F V3 non-NAb as an SPR probe. As found in previous SPR studies, this antibody reacted poorly with unmodified BG505 trimers (8, 28). Its binding was unchanged after cross-linking with EDC/EHS but modestly increased with GLA (Fig. 6B). After subsequent negative selection on the 19b column, there was no or negligible residual 39F binding to either GLA or EDC/NHS cross-linked trimers. In contrast, the V3/N332-glycan supersite bNAb PGT121 bound comparably well to all the trimer subpopulations tested. Thus, the SPR data are broadly consistent with the ELISA data and confirm that the cross-linked trimers have improved antigenicity properties after positive and negative affinity selection.

**FIG 7:**
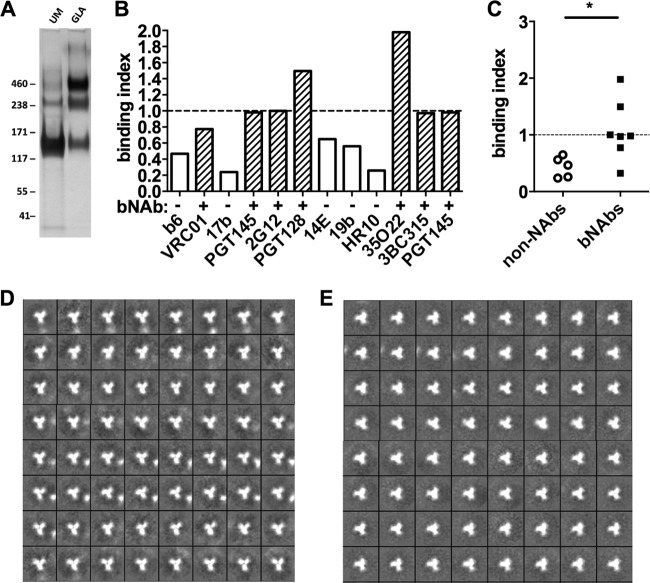
Properties of GLA cross-linked and PGT145 positively selected B41 SOSIP trimers. (A) SDS-PAGE analysis of unmodified (UM) and cross-linked (GLA) B41 trimers. (B) GLA cross-linked and PGT145-selected B41 was analyzed by capture ELISA. Binding indices were calculated as AUC (cross-linked)/AUC (unmodified). Values of <1 indicate there was a reduction in antibody binding; values of 1 (dotted line) represent no change in binding. Binding indices are shown for all MAbs tested. (C) The MAbs are grouped into neutralizing (bNAbs) and nonneutralizing (non-NAbs) categories; the dotted line represents no change in binding index. *, *P* < 0.05 Mann-Whitney U test. (D and E) 2D class averages from negative-stain EM analysis of unmodified (D) and GLA-treated and PGT145 selected (E) B41 trimers.

Finally, we tested cross-linking and PGT145 positive selection on a SOSIP trimer derived from the clade B strain B41. Like their BG505 counterparts, these trimers are fully native-like, but a variable proportion are in a partially open conformation while the rest adopt the fully closed state that dominates the BG505 trimer population (50). GLA cross-linking of B41 trimers using the conditions optimized for BG505 increased trimer stability in the SDS-PAGE assay, although, unlike BG505, substantial B41 monomer and dimer populations were also observed (Fig. 7A). As with BG505, the GLA cross-linked and PGT145 positively selected trimers were less reactive with non-NAbs, whereas bNAb binding was mostly preserved (Fig. 7B and C). A negative-stain EM analysis of the unmodified B41 trimers showed that >90% were in the fully closed conformation (Fig. 7D). This proportion is higher than previously reported (∼50%) for the same B41 trimers derived from the same stable CHO cell line and despite the use of the same 2G12 column purification strategy and identical EM methodology (50). The variation is likely to reflect the subtle influences of temperature and time on the position of equilibrium between the closed and partially open conformations (58). After cross-linking and PGT145 positive selection, >90% of the B41 trimers retained their closed morphology (Fig. 7E). Taken together, these data show that the cross-linking and positive-selection methodology developed using BG505 trimers can be applied to a second trimer from a different clade. It is reasonable to assume that the methods will prove to be highly generalizable.

## DISCUSSION

Soluble SOSIP.664 trimers are being evaluated as immunogens for NAb induction, since they structurally resemble the virion-associated Env spike and have native-like antigenic properties (12–14). In general, SOSIP.664 trimers bind bNAbs well and non-NAbs poorly or not at all (12, 13). However, like many Env vaccine candidates, the present generation of SOSIP.664 trimers can be improved, including from the perspectives of antigenicity and stability. Three areas relevant to the present work are the following. (i) Both membrane-anchored and soluble trimers are flexible structures that sample different conformational states (16, 18), which may influence the presentation of quaternary structure-dependent bNAb epitopes. (ii) The unliganded SOSIP.664 trimers can expose epitopes for non-NAbs under some conditions, of which V3 epitopes are the most antigenic and immunogenic (12, 13). (iii) The trimers bind CD4 and undergo conformational changes that expose non-NAb epitopes such as V3 and CD4i (12). Whether these properties of the present-generation trimers adversely affect their immunogenicity is presently under investigation, but it is prudent to consider that they may do so. Accordingly, we have evaluated whether chemical cross-linking can irreversibly trap BG505 trimers in certain conformational states and, in linked studies, whether positive and/or negative selection with bNAbs and non-NAbs, respectively, can isolate trimers with superior antigenic profiles in the absence and presence of CD4.

Cross-linking is a progressive chemical process affected by factors that include the cross-linker concentration, the temperature, and the pH of the reaction buffer. Our initial optimization studies allowed us to define two protocols whereby most quaternary bNAb epitopes were retained on the trimer, accompanied by a reduction in the binding of most non-NAbs. The two cross-linkers, GLA and EDC/NHS, had different effects on the trimer, most likely because the linking chemistries and the presence or absence of linker atoms differ. Thus, GLA cross-linking substantially reduced PGT145 binding to the BG505 trimer but preserved the PGT151 epitope, whereas EDC/NHS cross-linking had the converse effect. PGT151 and antibodies of the PGT145-class cross-compete for trimer binding via a noncompetitive bidirectional mechanism (13). Thus, it is possible that a cross-linked trimer conformer that efficiently displays the epitope for one of these quaternary structure-dependent bNAbs may be unable to simultaneously display the other. The basis of the differential effects on the two epitopes is not known but may reflect the different chemistries involved, i.e., amine-amine cross-linking for GLA and carboxyl-amine cross-linking for EDC/NHS. Since the individual protomers of the BG505 and B41 trimers are in close proximity, it is likely that interprotomer cross-links will be formed. This outcome would be fully consistent with the robust stability of the trimer under denaturing SDS-PAGE conditions and will probably contribute to the cross-linking-induced modifications to epitopes at the trimer apex where the three gp120 protomers form critical noncovalent contacts.

Both cross-linking methods trapped forms of Env that had nonnative morphology when viewed by EM and that also had some undesirable antigenic properties. The subsequent use of bNAb positive-selection columns, however, isolated fully native-like trimer subpopulations with improved properties when assessed by EM and SPR/ELISA. The antigenicity profiles of the positively selected, cross-linked trimers were similar but not identical to those of their unmodified counterparts. The subtle but reproducible differences in antibody reactivity may indicate that some or all of the cross-linked trimers are trapped in intermediate conformations and/or that the cross-linker has caused some perturbation to the local or global structure of the trimer. A more precise analysis of the cross-linked trimer is beyond the resolution of the methods used here but might be achievable if high-resolution cryo-EM structures could be obtained.

It may be possible to use the two cross-linkers to stabilize and differentially present epitope clusters in a targeted approach. For example, presenting the PGT145 epitope would most likely benefit from EDC cross-linking followed by PGT145 positive selection, whereas the PGT151 epitope would be best presented on GLA cross-linked trimers that were then positively selected with PGT151. An alternative approach might be to cross-link trimers bound to the selecting antibody on a column, followed by elution of the stabilized trimer. The reduced binding of CD4bs bNAbs to cross-linked trimers suggests either that this method may not be useful for presenting this epitope cluster to B cells or that positive enrichment using CD4bs bNAbs might be required to select for optimal antigenic forms. Similarly, the reduction in CD4 binding to cross-linked trimers may be relevant to overcoming any adverse effects arising from the CD4 induction of potentially distractive non-NAb epitopes *in vivo* (68). A related point is that, in principle, the immunogenicity of trimers could be decreased if they bound to CD4-expressing cells such as CD4^+^ T cells *in vivo* and were depleted before they had the opportunity to interact with B-cell receptors or be taken up by professional antigen-presenting cells. Negative selection of cross-linked trimers with a V3 non-NAb eliminated most V3 epitope-expressing conformers from the input population. As noted above, this finding may be useful if the strong immunogenicity of V3 adversely affects the presentation of bNAb epitopes to B cells or creates other interference effects on the induction of tier 2 NAb responses. Finally, cross-linked trimers may be generally more stable *in vivo*, including to proteases, thereby increasing their half-life. Whether any of these potential benefits do improve trimer immunogenicity can obviously be evaluated only in animal studies. These are now in progress but take >6 months to complete and analyze (14, 15).

In a recent study, full-length, membrane-anchored HIV-1 Env was cross-linked using homobifunctional amine-amine cross-linkers [bis(sulfosuccinimidyl)suberate (BS^3^) and 3,3-dithiobis(sulfosuccinimidylpropionate) (DTSSP)], extracted from the membrane using detergents, and then negatively depleted of aberrant forms by affinity chromatography with gp120- and gp41-specific non-NAbs. The endpoint was a trimeric Env preparation with improved antigenic characteristics compared to unselected forms in respect of its presentation of bNAb versus non-NAb epitopes (27). Protease digestion of misfolded forms of Env from the surface of virus-like particles is also being evaluated *in vitro* and *in vivo*, for broadly similar reasons (69, 70). Another approach involves targeted stabilization using structure-based design, which may create highly stable trimers without recourse to chemical modification or new trimer variants that could be further improved by cross-linking methodology (17, 71). Taken together with our own ongoing immunogenicity studies, these alternative approaches to a common problem may reveal whether the stabilization of Env proteins and/or the reduction in their presentation of non-NAb epitopes can focus and improve their immunogenicity from the perspective of bNAb induction.

Cross-linking of vaccine antigens is a proven method with an excellent safety record (72); it was first used to create inactivated polio vaccines and has since been used more widely (73). Although the traditional cross-linker has been formaldehyde, there are also extensive safety data on GLA-treated antigens from clinical trials in allergy immunotherapy (74, 75) and in the context of heart (76) and vascular (77) transplants. No clinical trials of EDC/NHS cross-linked proteins have been conducted to date, although its properties as a zero-length cross-linker that leaves no residual atoms associated with the immunogen suggest that its use is unlikely to have direct *in vivo* safety implications (78). Accordingly, any B-cell antigen that is conformationally or structurally unstable, including but not limited to HIV-1 Env trimers, may benefit from this cross-linking approach.

## Supplementary Material

Supplemental material
