# Supplementary material for: Chemical Cross-Linking Stabilizes Native-Like HIV-1 Envelope Glycoprotein Trimer Antigens
Source: J Virol. 2015 Dec 30;90(2):813–28. doi: 10.1128/JVI.01942-15 (PMC4702668; doi:10.1128/JVI.01942-15)
Supplement: Supplemental material [file supp_90_2_813__index.html]

Chemical Cross-Linking Stabilizes Native-Like HIV-1 Envelope Glycoprotein Trimer Antigens — Supplemental material 

# Chemical Cross-Linking Stabilizes Native-Like HIV-1 Envelope Glycoprotein Trimer Antigens

## Supplemental material

- Supplemental file 1 -

  Table S1 (Complete data set of binding indices.)

  Fig. 1 (Effects of positive and negative selection on antigenicity.)

  PDF, 612K
