# Supplementary material for: Chemical Cross-Linking Stabilizes Native-Like HIV-1 Envelope Glycoprotein Trimer Antigens
Source: J Virol. 2015 Dec 30;90(2):813–28. doi: 10.1128/JVI.01942-15 (PMC4702668; doi:10.1128/JVI.01942-15)
Supplement: Supplemental material [file JVI.01942-15_zjv999091153so1.pdf]

mAb	GLA	GLA +145	GLA +151	GLA -19b	EDC	EDC +145	EDC +315	EDC -19b
**15e**	0.21	0.78	0.08	0.04	0.06	0.11	0.10	0.00
**b6**	0.46	0.70	0.26	0.07	0.01	0.08	0.17	0.00
**B12**	0.28	0.57	0.25	0.09	0.01	0.05	0.09	0.01
**F105**	0.31	0.38	0.22	0.00	0.13	0.13	0.05	0.00
**HJ16**	0.84	1.05	0.99	0.77	0.87	0.88	0.86	0.71
**NIH45-46**	0.61	0.64	0.62	0.71	0.72	0.93	0.73	0.73
**VRC01**	0.86	0.85	0.92	0.95	0.39	0.39	0.38	0.31
**VRC03**	0.94	0.82	0.88	0.77	0.20	0.51	0.72	0.45
**17b**	0.12	0.15	0.07	0.03	0.07	0.03	0.01	0.00
**412D**	0.20	0.17	0.03	0.00	0.27	0.00	0.00	0.00
**A32**	0.15	0.27	0.06	0.01	0.02	0.05	0.09	0.02
**C11**	0.19	0.17	0.01	0.02	0.18	0.01	0.02	0.00
**CAP256-VRC26.08**	0.13	0.61	0.22	0.17	0.60	0.74	1.25	0.54
**CH01**	1.06	1.36	1.31	1.02	0.31	1.08	1.40	0.69
**PG16**	0.74	0.94	0.86	0.86	0.49	0.78	0.92	0.57
**PGDM1400**	0.36	0.72	0.46	0.43	0.67	0.79	0.80	0.70
**PGT145**	0.27	0.76	0.33	0.09	0.86	1.00	0.84	0.96
**SC258**	0.00	0.31	0.00	0.00	0.07	0.13	0.24	0.00
**2G12**	0.99	0.98	1.09	1.03	0.99	1.00	0.99	1.02
**PGT121**	0.90	0.93	1.03	1.05	0.87	0.94	0.92	1.02
**PGT128**	0.97	0.94	1.12	1.08	0.94	0.87	0.84	1.02
**PGT135**	1.01	1.00	1.02	0.96	0.86	1.02	0.98	0.97
**14E**	0.67	1.13	0.58	0.13	0.51	0.99	1.23	0.04
**19b**	0.56	3.49	0.67	0.08	0.79	1.80	3.06	0.05
**39F**	0.35	2.88	0.57	0.09	0.52	1.06	1.74	0.00
**HR10**	0.63	2.14	0.54	0.19	0.38	1.07	0.92	0.22

**HGN194**	0.63	1.40	0.58	0.19	0.48	0.73	0.89	0.23
**35O22**	0.80	0.67	0.74	0.60	0.70	0.81	0.81	0.73
**3BC176**	1.00	1.06	0.93	0.90	0.84	0.95	0.70	0.92
**3BC315**	1.04	0.96	1.05	0.95	0.60	0.66	0.33	0.42
**PGT151**	0.62	0.73	0.80	0.70	0.09	0.02	0.01	0.01
**7B2**	0.56	3.70	0.77	0.33	ND	1.24	2.21	0.00

Supplementary table 1: Complete dataset of binding indices. GLA (GLA) or EDC (EDC) cross-linked material was positively selected with PGT145 (+145), PGT151 (+151) or 3BC315 (+315) or negatively selected with 19b (-19b). Binding indices were calculated compared to unmodified trimers. Data shown are means of all (n=1 for EDC -19b, n=2-6 for all other antigens) independent experiments. Negative values are shown as 0.00. ND: not determined.

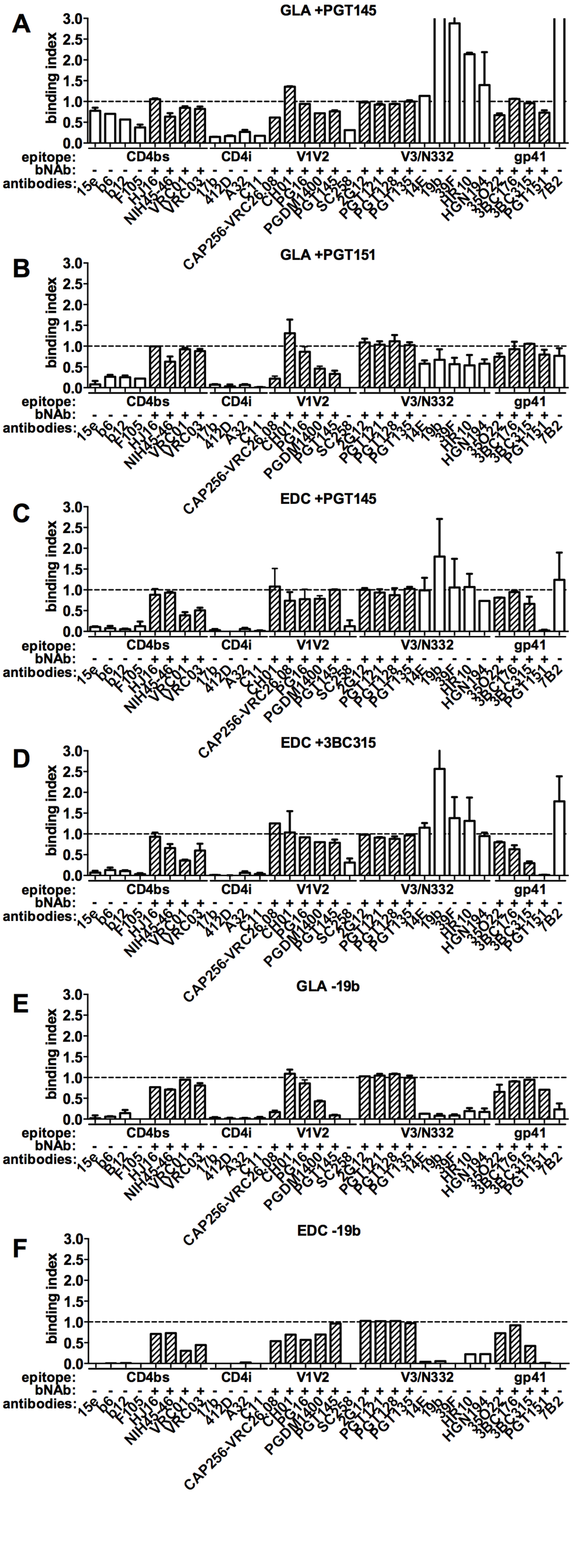
A
GLA +PGT145
binding index
3.0 2.5 2.0 1.5 1.0 0.5 0.0
epitope: CD4bs CD4i V1V2 V3/N332 gp41
bNAb:
antibodies: 15e b6 b12 F105 HJ16 NIH45-46 VRC01 VRC03 17b 412D A32 C11 CAP256-VRC26.08 CH01 PG16 PGDM1400 PGT145 SC258 2G12 PGT121 PGT128 PGT135 14E 19b 39F HR10 HGN194 35O22 3BC176 3BC315 PGT151 7B2
B
GLA +PGT151
C
EDC +PGT145
D
EDC +3BC315
E
GLA -19b
F
EDC -19b

Supplementary Fig. 1. Effects on antigenicity of positive and negative selection. BG505 was cross-linked with either GLA or EDC/NHS (EDC) and positively (+) or negatively (-) selected with the indicated antibody. Binding indices were calculated similarly to Fig. 2B and 4C. (**A**) GLA cross-linked trimers were positively selected on a PGT145 column. (**B**) GLA cross-linked trimers were positively selected on a PGT151 column. (**C**) EDC/NHS cross-linked trimers were positively selected on a PGT145 column. (**D**) EDC/NHS cross-linked trimers were positively selected on a 3BC315 column. (**E**) GLA cross-linked trimers were negatively selected on a 19b column. (**F**) EDC/NHS cross-linked trimers were negatively selected on a 19b column.
